# Spatial memory decline after masticatory deprivation and aging is associated with altered laminar distribution of CA1 astrocytes

**DOI:** 10.1186/1471-2202-13-23

**Published:** 2012-02-29

**Authors:** Marina Negrão Frota de Almeida, Fabíola de Carvalho Chaves de Siqueira Mendes, André Pinheiro Gurgel Felício, Manoela Falsoni, Márcia Lorena Ferreira de Andrade, João Bento-Torres, Pedro Fernando da Costa Vasconcelos, Victor Hugh Perry, Cristovam Wanderley Picanço-Diniz, Marcia Consentino Kronka Sosthenes

**Affiliations:** 1 Universidade Federal do Pará-UFPA, Instituto de Ciências Biológicas, Laboratório de Investigações em Neurodegeneração e Infecção, Hospital Universitário João de Barros Barreto, Belém, PA, Brazil; 2 Departamento de Arbovirologia e Febres Hemorrágicas, Instituto Evandro Chagas, IEC, Ananindeua, PA, Brazil; 3 Southampton Neuroscience Group, School of Biological Sciences, University of Southampton, Southampton SO16 7PX, UK; 4 Laboratório de Investigações em Neurodegeneração e Infecção, ICB/HUJBB/UFPA, Rua dos Mundurucus, 4487, Guamá, Belém, PA CEP:66073-000, Brazil

## Abstract

**Background:**

Chewing imbalances are associated with neurodegeneration and are risk factors for senile dementia in humans and memory deficits in experimental animals. We investigated the impact of long-term reduced mastication on spatial memory in young, mature and aged female albino Swiss mice by stereological analysis of the laminar distribution of CA1 astrocytes. A soft diet (SD) was used to reduce mastication in the experimental group, whereas the control group was fed a hard diet (HD). Assays were performed in 3-, 6- and 18-month-old SD and HD mice.

**Results:**

Eating a SD variably affected the number of astrocytes in the CA1 hippocampal field, and SD mice performed worse on water maze memory tests than HD mice. Three-month-old mice in both groups could remember/find a hidden platform in the water maze. However, 6-month-old SD mice, but not HD mice, exhibited significant spatial memory dysfunction. Both SD and HD 18-month-old mice showed spatial memory decline. Older SD mice had astrocyte hyperplasia in the strata pyramidale and oriens compared to 6-month-old mice. Aging induced astrocyte hypoplasia at 18 months in the lacunosum-moleculare layer of HD mice.

**Conclusions:**

Taken together, these results suggest that the impaired spatial learning and memory induced by masticatory deprivation and aging may be associated with altered astrocyte laminar distribution and number in the CA1 hippocampal field. The underlying molecular mechanisms are unknown and merit further investigation.

## Background

Previous studies have established an association between chewing activity and cognition [1-3]. The systemic effects of long-term masticatory imbalances are associated with neurodegeneration and are a risk factor for senile dementia in humans [4] and memory deficits in experimental animals [5]. To investigate the impact of masticatory imbalances on various activities and physiological factors, experimental masticatory deprivation has been modelled experimentally in animals using a modified diet [6], molar removal [7,8], or occlusion disharmony modelled by "bite-raised" condition [9,10]. These approaches revealed that masticatory dysfunction reduces spatial learning and memory in water maze tests in rats [8,11] and mice [11-13], and that these deficits seem to increase with aging and time after tooth loss [8]. Induced molarless subjects, revealed reduced neurogenesis in young rats [14], and there is a loss of astrocytes, neurons and dendritic spines in the hippocampus of aged rats and mice [7,14,15]. Young mice fed a soft diet show reduced neurogenesis and BDNF levels [16,17], whereas progressive synaptic reduction and pyramidal neuron losses are observed in aged mice [6,18].

Masticatory deprivation seems to affect mainly the hippocampus of all ages, and young, middle-aged and senile mice subjected to masticatory imbalances show a decreased number of neurons in CA1 [11,18] and CA3 [18] and an increased number of hypertrophic astrocytes in CA1 [8]. All of these changes seem to be aggravated by aging [15] and time after tooth loss [11], suggesting additive effects. However, to our knowledge, no stereological analysis has been conducted to date that explores the long-term glial changes induced by a soft diet regime imposed early in life.

Notably, cerebral blood flow in various areas of the brain is affected by mastication [19-21], and astrocytes are likely to play a key role in regulating cerebral blood flow [22-25]. In particular, the regulation of blood flow seem to be mediated by astrocyte calcium (Ca^2+^) signalling, which may induce both arteriolar dilation and constriction as a function of calcium concentration [25].

Since previous results demonstrated that aging affects spatial memory and astrocytes in the dentate gyrus [26] and hippocampus [27] in a laminar-dependent fashion, we tested the hypothesis that long-term masticatory deprivation and aging have additive effects on spatial memory and on the laminar distribution of CA1 astrocytes as assessed by an optical fractionator, which is an unbiased stereological analysis method.

## Results

### Masticatory reduction and water maze tests in 3-, 6- and 18- month-old mice

**Figure 1:**
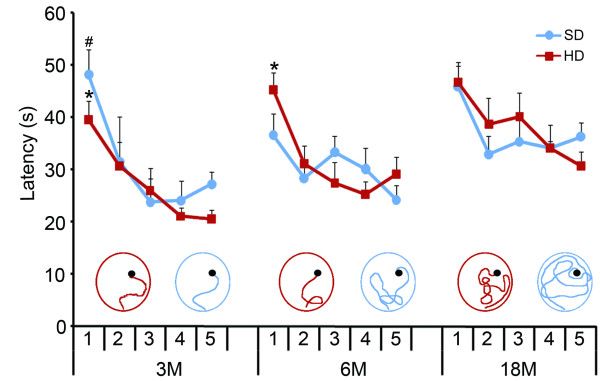
**Performances on the water maze tests applied to 3-, 6- and 18-month old mice were compared by determining the escape latencies on five test days**. Three-month-old SD and HD mice showed similar latencies, while the escape latencies of 6-month-old HD mice were significantly shorter than those of SD age-matched mice. SD and HD 18-month-old mice did not show any significant change in escape latencies. Swimming tracking analysis revealed that SD had longer trajectories as compared to age-matched HD mice, particularly at 6 months of age (inset). HD = hard diet; SD = soft diet (* = *p *< 0.05 for the HD group. # = *p *< 0.05 for the SD group)

Figure 1 shows a graphic representation of the escape latencies for water maze tests conducted on five consecutive days on mice that were 3-, 6- and 18-months old. Three-month-old SD and HD mice showed similar performance on the water maze test. Indeed, both groups showed significant decreases in the escape latencies on the 3rd day test as compared to the 1st day test (Figure 1). For the 6-month-old mouse groups, only the HD mice were able to significantly reduce latency values over the 5-day period. Finally, 18-month-old mice from both the SD and HD groups were unable to reduce escape latency during the 5-day testing period, and there were no significant differences in the escape latencies on the 1st and the 5th test days in either group. Analysis of the swim paths revealed that the SD group had, overall, longer and more erratic trajectories compared to age-matched HD mice (Figure 1, inset). Neither masticatory deprivation nor aging had a significant influence on swimming speed, suggesting that the negative effects on latency were due to impaired spatial memory rather than to alter swimming per se.

**Figure 2:**
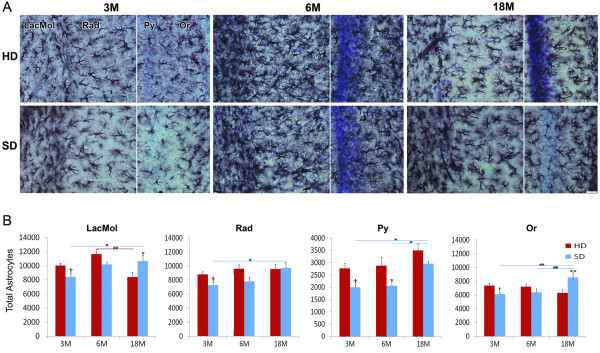
**Laminar distribution and graphic representations of quantitative data of astrocytes in the CA1 hippocampal field**. (**A**): Photomicrographs of CA1 layers in hippocampal sections from 3-, 6- and 18-month-old SD and HD mice. These sections were representative of mice that had astrocyte levels that were close to the mean values for each group. (**B**): The mean number and standard errors (s.e.m.) of stereological estimates of the total number of astrocytes for each CA1 layer in mice of the indicated age. (*) = Statistically significant differences (two tailed *t*-test; *p *< 0.05) between HD and SD age-matched mice. (#) = Statistically significant differences between SD and HD mice of different ages. LacMol. = stratum lacunosum-moleculare; Rad = stratum radiatum; Py = stratum pyramidale; Or = stratum oriens; HD = hard diet; SD = soft diet; 3 M, 6 M and 18 M indicate 3-month-old, 6-month-old and 18-month-old mice, respectively. Scale bar = 25 μm

Figure 2 shows photomicrographs of CA1 from the indicated mouse groups as well as the laminar distribution of astrocytes; graphs of the mean number and standard errors for the estimated number of astrocytes in the strata lacunosum-moleculare, radiatum, pyramidale and oriens are also shown. (For detailed data, see Additional file 1: Table S1, Additional file 2: Table S2, Additional file 3: Table S3 and Additional file 4: Table S4, Additional file 5: Table S5, Additional file 6: Table S6, Additional file 7: Table S7 and Additional file 8: Table S8). The representative sections chosen for Figure 2 were from mice that had an estimated number of astrocytes that was close to the mean group value. It is clear in the photomicrographs that there are qualitative age-related changes in the control "normal diet" HD group. From 3 months to 6 months, there was a marked increase in branching density associated with changes in the number and intensity of glial fibrillary acidic protein (GFAP) immunolabeled branches. There was strong GFAP immunolabeling near the arteries in the hippocampal fissure, suggesting a role for astrocytes in local blood flow in these layers of CA1. However, by 18 months there was a clear reduction in branching density and marked atrophy of the branching as revealed by GFAP immunolabeling. Changes in astrocyte number were quantified, revealing that there were significantly fewer astrocytes in 3-month-old SD mice compared to 3-month-old HD mice in all CA1 layers (two-tail *t*-test, *p *< 0.05). However, 6-month-old mice only showed SD vs. HD differences in the stratum pyramidale (two-way ANOVA, Bonferroni post-hoc tests, *p *< 0.05). In 18-month-old mice, there was an increase in the number of astrocytes in the strata lacunosum-molecular and oriens of HD mice compared to SD mice (two-way ANOVA, Bonferroni post-hoc tests, *p *< 0.05). Aging-induced astrocytosis in all CA1 layers was evident in 18-month-old SD mice compared with 3-month-old mice. However, the only significant differences noted in 6-month-old SD mice compared with 18-month-old SD mice were in the strata pyramidale and oriens. Thus, aging and diet seemed to induce additive astrocytosis, particularly in the strata lacunosum moleculare and oriens (two-way ANOVA, *p *< 0.01). Overall, the main effects of the SD were to reduce the number of astrocytes at 3 and 6 months and then to prevent further age-related losses at 18 months in the lacunosum-moleculare layer.

The HD and SD groups showed significant differences in the total number of astrocytes in CA1 at 3 months but not at 6 months or 18 months. Indeed, SD mice had, on average, fewer astrocytes at 3 months (23,839 ± 653) compared to age-matched HD mice (28,999 ± 807), (*p *= 0.002). However, there were no differences in the total number of astrocytes in SD vs. HD mice at 6 months (26,477 ± 1572 vs. 31,440 ± 1613) or 18 months (31901 ± 2046 vs. 27780 ± 1965), (*p *> 0.05), respectively. Aging only induced significant astrocytosis in SD mice at 18 months compared with SD mice at 3 months and 6 months.

## Discussion

The results of the present study demonstrated that 24-hour-per-day masticatory deprivation had differential laminar effects on the number of astrocytes in CA1 and on water maze memory test performance in 3-, 6- and 18-month-old female albino Swiss mice. We suggest that masticatory deprivation can induce abnormal cognitive development and may enhance changes in aging astrocytes in CA1.

### Hippocampal astrocytes, water maze performances and reduced mastication

In the present report, water maze tests revealed that after 6 or 18 months of masticatory deprivation, experimental SD mice could not learn and remember the position of a hidden platform; in contrast, control 6-month old HD mice could learn and remember the position of a hidden platform, whereas 18-month-old mice from both groups SD and HD were unable to reduce escape latency during the 5-day testing period. There were no significant differences in the swimming speeds of young (3-month-old), mature (6-month-old) and aged (18-month-old) mice in either the SD or the HD group, suggesting that spatial memory impairment, rather than motivation or physical performance, was a more likely explanation of the differences in the water maze performance. In parallel with memory dysfunction, there were significant changes in the laminar distribution of astrocytes in CA1. A continuous SD that started early in life (21^st ^postnatal day) induced astrocytic hypoplasia at 3 months in all layers and in the pyramidal layer at 6 months as compared to age-matched HD mice. Compared to 18-month-old SD mice and 6-month-old HD mice, 18-month-old HD mice showed hypoplasia in the strata lacunosum moleculare. In addition, 18-month-old SD mice showed significantly more astrocytosis than 3-month-old and 6-month-old SD mice. Taken together, the results suggest that masticatory deprivation induced abnormal cognitive development and may have enhanced changes in aging astrocytes in CA1.

Although there were no learning and memory differences in the water maze test [28] after 3 months of masticatory deprivation, the laminar distribution of CA1 astrocytes started to change when the SD mice were 3 months old and became especially severe after six months or more of masticatory deprivation. Since spatial memory and learning were investigated previously only in adult (6-month-old) but not in aged (18-month-old) albino Swiss mice [29-31], this is the first report to assess the effects of long-term masticatory deprivation (using SD to model deprivation) on astrocytes by stereological analysis of CA1.

The accelerated cognitive decline observed in SD mice was not directly correlated with time-specific changes in astrocyte number in any CA1 layer. Thus, we investigated whether there were morphological changes (e.g. evident shrinkage of astrocyte arbors) that affected the astrocyte network. Long-term potentiation (LTP), considered the neurophysiological basis for learning and memory, is facilitated by brain-derived neurotrophic factor (BDNF) in both young and aged mice. However, in aged mice the effects of exogenous BDNF on LTP does not translate into improved spatial memory [32]. Interestingly, BDNF expression in the hippocampus of C57Bl6 mice is reduced under SD as compared to HD at 3 and 6 months [16]; in parallel, there is reduced neurogenesis in SD mice of the same ages [17]. We also observed a reduction in the number of astrocytes at 3 and 6 months in SD mice vs. HD mice. Notably, astrocytes synthesize and release BDNF, which is critical for experience-dependent synaptic plasticity in the mature brain [33], and there is a reduction of hippocampal BDNF levels in aged mice, with deleterious consequences for synaptic plasticity and spatial memory [34].

In the present report, there were significantly more astrocytes in the stratum oriens at 18 months in SD mice compared to HD mice. The combination of aging and masticatory deprivation affects the stratum oriens in an additive manner, reducing both dendritic spine density [7] and synaptic density [6] and significantly reducing cell size in SD and HD groups. These results suggest that astrocytic changes induced by aging and masticatory deprivation are greater in the strata lacunosum-moleculare and radiatum than changes in the strata pyramidale and oriens.

Many studies show that the CA1 is important in signalling pathways that are key for learning and memory (reviewed in [35]). Specifically, the lacunosum-moleculare layer is the target of the temporoammonic pathway, which originates in the entorhinal cortex and creates excitatory glutamatergic synapses with distal pyramidal dendrites in CA1 in mice [36] and rats [37,38]. Temporoammonic synapses are associated with theta oscillations and late-phase LTP and long-term memory consolidation [39-41]. In addition, astrocyte calcium signals at Schaffer collateral to CA1 pyramidal cell synapses correlate with the number of activated synapses [42], demonstrating the direct participation of astrocytes in the hippocampal circuits that are involved in spatial memory. The presence of synaptic potentiation has been described previously to occur via astrocytic glutamate exocytosis at the entorhinal-to-dentate granular cells (the perforant pathway) [43,44] and Schaffer collaterals [45]. In this way, astrocytes play a critical role in memory functioning and LTP and contribute to synaptic tuning in the hippocampus [44].

Astrocytes in the hippocampus seem particularly sensitive to age-related changes [46-48] and can be impaired by structural/functional changes induced by masticatory imbalances (see, for example, [5-7]). Indeed, impairment of spatial memory is reported to occur in aged mice when the molar teeth are extracted or cut when the mice are young [11] as well as in adult rats that are fed a SD after the weaning period [6]. In these studies, both neuronal density in the CA1 hippocampus [11] and synaptic formation in the hippocampus and the parietal cortex [6] were reported to decrease. In agreement with these studies, tooth loss and the resulting masticatory alterations leads to a reduction in the number of ChAT-positive neurons in the Broca diagonal band and medial septum, resulting in a decrease of acetylcholine levels in the hippocampus [49,50].

Most of the earlier studies of the impact of reduced mastication on the hippocampus focused on neuronal changes. To our knowledge, there are only a few reports that employed GFAP-immunolabeling of the CA1 hippocampal field, and all such studies were restricted to aged SAMP8 mice. These studies revealed that the molarless condition enhances the age-dependent increase in the density and hypertrophy of astrocytes, and that these effects increase the longer the molarless condition persists [8,15].

The notion that aging and masticatory deprivation may cause similar cellular and molecular alterations in the hippocampus is supported by other studies (reviewed in [51]). Indeed astrocyte hypertrophy has been reported previously in the CA1 hippocampal field [8,15], suggesting that these cells may increase their production of pro-inflammatory cytokines in response to inflammation. Similar changes have been described in the senile hippocampus, where a more reactive astrocyte phenotype is expressed during aging, even in the absence of neurological disease, as part of an increased and maintained pro-inflammatory profile that may be associated with cognitive dysfunction (reviewed in [52]).

It is unclear why the number of astrocytes in the stratum lacunosum-moleculare was reduced in 18-month-old HD mice compared to 6-month-old HD mice. However, since BDNF regulates gliogenesis, it could be that the reduced levels of BDNF induced by aging contributed to the decrease in the number of neurotrophic astrocytes [53]. In addition, we suggest that the astrocytosis observed in the stratum oriens of 18-month-old SD mice is not dependent on BDNF but, rather, is part of the inflammatory aging process [52]. Although the present investigation is related to chronic stress (since there was a long period of masticatory deprivation), previous results regarding the acute stress response during immobilization revealed that biting can restore the BDNF mRNA levels that are reduced by stress [54]. Taken together, the results suggest that a decrease in masticatory activity along with aging may promote the observed differential laminar effects on CA1 astrocytic plasticity.

### Astrocytes, aging and CA1 blood flow changes

The strong GFAP immunolabeling we observed near the arteries in the hippocampal fissure suggests a role for astrocytes in local blood flow regulation in these layers of the CA1 and dentate gyrus. Indeed, there is some evidence that astrocytes are involved in hippocampal neurovascular control [24]. Astrocytes contribute to local blood flow in the hippocampus, and we observed a statistically significant reduction in the number of GFAP-immunolabeled cells in the lacunosum moleculare of CA1 in HD mice late in life; thus, we predict that hippocampal blood flow is altered in 18-month-old mice. The decrease in GFAP immunolabeling may be secondary to a reduction of neuronal activity induced by aging in the temporoammonic pathway in mice [36,55]. However, since the astrocytic atrophy in SD mice seemed more severe than in HD mice, an alternative interpretation is that aging and a SD could be related to the decrease in functional memory at 18 months. Since we did not investigate possible correlations between the density of GFAP-positive processes and astrocyte function, this remains a key issue that merits further investigation. Similar alterations occur in the human hippocampus [56], and a major disturbance in cerebral blood flow late in life that is associated with axon-glial disruption could link vascular disease and chronic neurodegenerative diseases that are associated with aging [57]. Since tooth loss [58] and neurovascular dysfunction [59] seem to predict poor cognitive function in older humans, it is reasonable to hypothesize that masticatory deprivation in humans contributes, at least in part, to spatial memory dysfunction in aging [60]. Changes in hippocampal astrocytes may be associated with this as well.

### Astrocytic changes, corticosteroids and masticatory stress

Active mastication can rescue the stress-attenuated hippocampal memory process in animals by suppressing endocrinological and autonomic stress responses [51]. Indeed, previous studies of molarless mice showed plasma corticosterone levels that were significantly greater than those in molar-intact control mice. In addition, pretreatment with suppressors of stress-induced increases in plasma corticosterone levels prevented the molarless-induced increase in plasma corticosterone levels [61]. In addition, elevated corticosterone levels suppress synaptic plasticity in the hippocampus [62], and mastication suppresses the stress-activated expression of corticotropin-releasing factor (CRF)[63]. Astrocytic plasticity in the hippocampus may also be affected by corticosteroids due to changes in the number of astrocytes [64,65] or to changes in astrocyte morphology and function [66]. In particular, corticosterone increases the number of astrocytes in CA1 in a dose-dependent fashion [64].

In this study, masticatory deprivation-induced stress may have altered the plasma glucocorticoid levels, and this may in turn have affected astrocytic plasticity. We did not measure plasma corticosteroid levels; therefore, we cannot exclude the possibility that altered levels of corticosteroids might explain the observed effects of masticatory stress. However, there is currently no information about changes in glucocorticoid levels that might be induced by a SD, so it is difficult to evaluate this possibility.

### Hormones and astrocytes

It has now evident that ovarian steroids influence cognition and aging [67,68] and that hormonal cycle disruption late in life is associated with memory impairment [69,70]. In addition, females and males show striking differences in cognitive decline associated with aging, mainly in visuospatial abilities [71]. Because females are more susceptible to the cognitive decline that accompanies aging, it would be expected that the additive effects of estropause and aging would be greater than in males. Interestingly, aged C57Bl6J female mice have 18.3% more astrocytes than young female mice and 32% more than aged male mice [72]. Since the SD and HD mice in the present report were age-matched, it is reasonable to assume that mice in both 18-month-old groups were in estropause and to hypothesize that the significant differences in spatial memory and CA1 astrocytes detected in these mice were due to differences in diet as well as to aging.

### Non-stereological technical limitations

Estimations of the number of objects of interest in stereological studies can vary among studies. To detect the effects of variations in the criteria for identifying objects of interest in this study, we looked at the polymorphic layer of the dentate gyrus in aged female albino Swiss mice, which was previously described as having an increased number of astrocytes (aged = 10,232 ± 1325 vs. young = 6440 ± 1085, *p *< 0.05) [73]. As expected, we found that there were more astrocytes in the polymorphic layer of aged HD mice (18-month-old HD = 11,101 ± 1904) compared to young mice (6-month-old HD = 8162 ± 1354; *p *< 0.05).

The level of acceptable errors for stereological estimations was defined as the ratio between the intrinsic error introduced by the methodology and the coefficient of variation [74,75]. The coefficient of error (CE) reflects the accuracy of the cell number estimates, and CE values ≤ 0.05 were deemed appropriate for the present study since the variance introduced by the estimation procedure contributed little to the observed group variance. As a result, variations associated with non-biological sources were reduced to acceptable levels in the present report [72,75].

## Conclusions

Taken together, the results of this study suggest that the abnormal spatial learning and memory induced by long-term masticatory deprivation and aging may be associated with altered laminar distribution and a reduced number of astrocytes in CA1, as demonstrated by stereological analysis. The molecular mechanisms remain to be elucidated.

## Methods

The animals were treated in accordance with the guidelines published by the NIH (*Guide for the Care and Use of Laboratory Animals*), and all experimental procedures were approved prior to study initiation by the Ethics Committee on Experimental Animal Research (Institute of Biological Sciences, Federal University of Pará, Brazil).

### Animals and experimental groups

In this study, 66 mice were housed in groups of 6 in standard plastic cages (32 × 39 × 16.5 cm) covered by metal grids until they were sacrificed. All behavioural assays were performed using 3-, 6- and 18-month old mice. The reduced mastication condition began on the 21st post-natal day by the introduction of a powder diet (SD mice); control mice (HD mice) were fed a pellet diet. Mice had free access to food and water and were raised under controlled room temperature (23 ± 1°C) and 12-h light-dark cycles. There were no significant differences in body weight in the SD and HD experimental groups (measured the day of sacrifice), suggesting that the nutritional value of SD and HD was the same.

Compared to the groups of young mice (3- and 6-month-old mice), aging was associated with significant number of deaths. Although we have not found any significant differences in body weight or behaviour before deaths there was greater mortality in the 18-month-old SD group compared to the 18-month-old HD group.

### Behavioural tests

All tests were recorded with a webcam, and images were analysed with a computer program to score mouse performance in open field and water maze tests (ANY-maze Tracking System, Stöelting). Computer analysis was performed off-line. Behavioural tests were performed during the light cycle (08 h-12 h).

#### Water maze test

3-month-old (HD3M, n = 12; SD3M, n = 12), 6-month-old (HD6M, n = 12; SD6M, n = 12) and 18-month-old (HD18M, n = 13; SD18M, n = 7) mice were trained in a water maze adapted for mouse dimensions. The circular pool and platform were 94 and 14 cm in diameter, respectively, and the platform was 1 cm below the water surface. To hide the black platform, the pool was filled with black water (22 ± 2°C) coloured with a non-toxic dye. In each trial, the mice were allowed 60 s to find the hidden platform; trials were separated by intervals of 30 s. The task was considered complete when animals found and remained on the platform for 10 s. The first day of water maze training allowed the animals to adapt to the aquatic labyrinth. On the remaining four days, animals were tested in three trials once per day. We recorded the escape latency, distance travelled, average swimming speed and trajectories for each mouse. The visual cues outside the water maze were stable on all training days. The learning rate for the water maze was calculated by comparing results on the 1^st ^and 5^th ^test days. All groups were compared using one-way ANOVA, the Bonferroni *a priori *test or two-way ANOVA followed by Bonferroni post-tests, with intra- or between-group differences considered significant at a 95% confidence level (*p *< 0.05).

### Perfusion and histology procedures

After the 5-day water maze behavioural tests, the mice were weighed and sacrificed with an overdose of ketamine (100 mg/kg) and xylazine (10 mg/kg) (Konig Laboratories). They were then perfused transcardially with heparinized saline for 10 min, followed by an aldehyde fixative (4% paraformaldehyde in 0.1 M phosphate buffer, pH 7.2-7.4) for 30 min. All chemicals were purchased from Sigma (São Paulo, Brazil). After perfusion and craniotomy, the brains were removed and cut on a vibratome at a 70-μm thickness. One of each 5 sections was used for GFAP detection using free-floating immunohistochemistry. Free-floating sections were rinsed once in 0.1 M phosphate buffer, transferred to 0.2 M boric acid pH 9.0, heated to 65-70°C for 1 h and then washed 3 × 5 min in PBST (5%). The sections were incubated in a 1% hydrogen peroxide solution in methanol under constant and gentle shaking for 10 min and rinsed 2 × 2 min in 0.1 M PBS. The sections were then blocked with immunoglobulin for 1 h, following the instructions for the Mouse-on-Mouse Immunodetection kit (M.O.M. kit, Vector Laboratories, USA). Blocking was followed by washing (3 × 2 min) in PBS. Sections were incubated in a working solution of protein concentrate for 5 min, then incubated with monoclonal mouse anti-GFAP primary antibody (MAB360, Chemicon International, USA) diluted 1:800 in protein concentrate solution (M.O.M. kit) at 4°C for 3 days with continuous gentle agitation. Next, the sections were washed (3 × 2 min) in PBS and incubated for 20 h with biotinylated horse anti-mouse secondary antibody (M.O.M. kit), diluted 1:100 in PBS. After washing (3 × 2 min) in PBS, sections were transferred to an avidin-biotin-peroxidase complex solution (ABC, Vector Laboratories, USA, 1:200) for 1.5 h, washed (3 × 2 min) in 0.1 M PB, and processed using the glucose oxidase-DAB-nickel method and peroxidase histochemistry (Shu et al. 1988).

The reaction was interrupted when fine astrocytic branches were detected under the microscope. Sections were rinsed (4 × 5 min) in 0.1 M PBS, mounted on gelatinized slides, dehydrated in alcohol and xylene, and topped by a coverslip with Enthelan (Merck).

### Photomicrograph documentation and processing

Digital photomicrographs were taken with a digital camera (Microfire, Optronics, CA, USA) coupled to a Nikon microscope (Optiphot-2, NY, USA). The digital photomicrographs were processed with Adobe Photoshop 7.0.1 C.S.2 software (San Jose, CA, USA) for scaling; adjustments to the levels of brightness and contrast were applied to the entire image. The selected micrographs display representative sections from each experimental group i.e. the astrocyte number in each region of interest was close to the mean value for that region.

### Microscopy and optical fractionator

Details of the optical fractionator methodology and experimental parameters are described in online supplementary material (Additional file 5: Table S5, Additional file 6: Table S6, Additional file 7: Table S7 and Additional file 8: Table S8). In brief, we delineated the region and layers of CA1 at all levels in the histological sections, digitizing directly from sections using a low power 3.2× objective on a Optiphot-2 microscope (Nikon, Japan) equipped with a motorized stage (MAC200, Ludl Electronic Products, Hawthorne, NY, USA). This system was coupled to a computer running Stereoinvestigator software (MicroBrightField, Williston, VT, USA), which was used to store and analyse the x, y and z coordinates of digitized points. To detect and count unambiguously the objects of interest in the dissector probe, the low power objective was replaced by a 60× oil immersion Plan apochromatic objective (NIKON, NA 1.4) to count astrocytes.

### Statistical analysis

All groups of animals were tested for statistical normality. Possible outliers identified based on standard deviations were eliminated from the data set. Parametric statistical analysis was used to assess the level of significance of the results of behavioural tests, one-way ANOVA, and the Bonferroni *a priori *test. The significance level for statistical differences was set at alpha < 0.05 (i.e. at a 95% confidence level). Results from the optical fractionator were analysed using two-way ANOVA. A two-tailed *t*-test was used to compare age-matched groups with different diets (SD vs. HD). Statistical analyses were performed using BioEstat^® ^5.0, Statistica for Windows^® ^version 5.0 A and GraphPad Prism 5 for Windows^®^.

## Competing interests

The authors declare that they have no competing interests.

## Authors' contributions

MNFA, FCCSM, APGF, MF, MLFA, CWPD and MCKS conceived the study, participated in the experiment design and drafted the manuscript. MNFA, FCCSM, APGF, MF, MLFA, CWPD and MCKS performed the experiments and analysed and interpreted the data. JBT, PFCV, VHP, CWPD and MCKS participated in the data analysis, were involved in drafting the manuscript, and made important intellectual contributions. All authors read and approved the final manuscript.

## Supplementary Material

Addtional file 1 **Table S1**. Estimated Unilateral Number of Astrocytes (N) With the Coefficient of Error (CE) for the Stratum Lacunosum-Moleculare of CA1 of 3-, 6- and 18-Month-Old Female Albino Swiss Mice Fed A Hard Diet (HD) or Soft Diet (SD). Click here for file

Additional file 2 **Table S2**. Estimated Unilateral Number of Astrocytes (N) With the Coefficient of Error (CE) for the Stratum Radiatum of CA1 of 3-, 6- and 18-Month-Old Female Albino Swiss Mice Fed A Hard Diet (HD) or Soft Diet (SD). Click here for file

Additional file 3 **Table S3**. Estimated Unilateral Number of Astrocytes (N) With the Coefficient of Error (CE) for the Stratum Pyramidale of CA1 of 3-, 6- and 18-Month-Old Female Albino Swiss Mice Fed A Hard Diet (HD) or Soft Diet (SD). Click here for file

Additional file 4 **Table S4**. Estimated Unilateral Number of Astrocytes (N) With the Coefficient of Error (CE) for the Stratum Oriens of CA1 of 3-, 6- and 18-Month-Old Female Albino Swiss Mice Fed A Hard Diet (HD) or Soft Diet (SD). Click here for file

Additional file 5 **Table S5**. Experimental parameters and optical fractionator counting results in the stratum lacunosum-moleculare of the CA1 of 3-, 6- and 18-month-old female albino Swiss mice fed a hard diet (HD) or soft diet (SD). Click here for file

Additional file 6 **Table S6**. Experimental Parameters and Optical Fractionator Counting Results in the Stratum Radiatum of CA1 of 3-, 6- and 18-Month-Old Female Albino Swiss Mice Fed A Hard Diet (HD) or Soft Diet (SD). Click here for file

Additional file 7 **Table S7**. Experimental Parameters and Optical Fractionator Counting Results in the Stratum Pyramidale of CA1 of 3-, 6- and 18-Month-Old Female Albino Swiss Mice Fed A Hard Diet (HD) or Soft Diet (SD). Click here for file

Additional file 8 **Table S8**. Experimental Parameters and Optical Fractionator Counting Results in the Stratum Oriens of CA1 of 3-, 6- and 18-Month-Old Female Albino Swiss Mice Fed A Hard Diet (HD) or Soft Diet (SD). Click here for file
