# Supplementary material for: Spatial memory decline after masticatory deprivation and aging is associated with altered laminar distribution of CA1 astrocytes
Source: BMC Neurosci. 2012 Feb 29;13:23. doi: 10.1186/1471-2202-13-23 (PMC3355053; doi:10.1186/1471-2202-13-23)
Supplement: Addtional file 1 — Table S1. Estimated Unilateral Number of Astrocytes (N) With the Coefficient of Error (CE) for the Stratum Lacunosum-Moleculare of CA1 of 3-, 6- and 18-Month-Old Female Albino Swiss Mice Fed A Hard Diet (HD) or Soft Diet (SD). [file 1471-2202-13-23-S1.PDF]

Table S1. Experimental Parameters and Optical Fractionator Counting Results in the Stratum Lacunosum Moleculare of CA1 of 3-, 6-, and 18-Month-Old Female Albino Swiss Mice Fed With Hard Diet (HD) or Soft Diet (SD).

***Lacunosum-Moleculare* - CA1**								
**Subjects[a]**	**a(frame) (μm^2)**	**A(x,y step) (μm^2)**	**asf**	**tsf**	**ssf**	**N. of couting frames**	**N. of sections**	**ΣQ$^-$**
***Hard Diet* / 3M**								
HD 3M Animal 1	80 x 80	80 x 80	1	0.34 ± 0.005	1/6	140	5	563
HD 3M Animal 2	80 x 80	80 x 80	1	0.30 ± 0.006	1/6	136	5	541
HD 3M Animal 3	80 x 80	80 x 80	1	0.27 ± 0.010	1/6	123	6	415
HD 3M Animal 4	80 x 80	80 x 80	1	0.30 ± 0.006	1/6	129	5	528
***Hard Diet* / 6M**								
HD 6M Animal 1	80 x 80	80 x 80	1	0.32 ± 0.010	1/6	125	5	532
HD 6M Animal 2	80 x 80	80 x 80	1	0.40 ± 0.008	1/6	161	6	801
HD 6M Animal 3	80 x 80	80 x 80	1	0.38 ± 0.003	1/6	156	6	763
HD 6M Animal 4	80 x 80	80 x 80	1	0.33 ± 0.005	1/6	152	6	711
***Hard Diet* / 18M**								
HD 18M Animal 1	80 x 80	80 x 80	1	0.29 ± 0.003	1/6	137	6	438
HD 18M Animal 2	80 x 80	80 x 80	1	0.30 ± 0.003	1/6	148	6	454
HD 18M Animal 3	80 x 80	80 x 80	1	0.30 ± 0.006	1/6	116	5	339
HD 18M Animal 4	80 x 80	80 x 80	1	0.29 ± 0.004	1/6	128	5	459
***Soft Diet* / 3M**								
SD 3M Animal 1	80 x 80	80 x 80	1	0.33 ± 0.006	1/6	137	6	466
SD 3M Animal 2	80 x 80	80 x 80	1	0.35 ± 0.007	1/6	129	5	483

SD 3M Animal 3	80 x 80	80 x 80	1	0.32 ± 0.004	1/6	125	5	441
SD 3M Animal 4	80 x 80	80 x 80	1	0.34 ± 0.010	1/6	102	5	579
SD 3M Animal 5	80 x 80	80 x 80	1	0.26 ± 0.006	1/6	101	5	421
***Soft Diet* / 6M**								
SD 6M Animal 1	80 x 80	80 x 80	1	0.35 ± 0.015	1/6	147	5	679
SD 6M Animal 2	80 x 80	80 x 80	1	0.37 ± 0.013	1/6	134	5	602
SD 6M Animal 3	80 x 80	80 x 80	1	0.33 ± 0.014	1/6	123	5	522
SD 6M Animal 4	80 x 80	80 x 80	1	0.28 ± 0.012	1/6	132	6	493
SD 6M Animal 5	80 x 80	80 x 80	1	0.29 ± 0.008	1/6	115	5	458
***Soft Diet* / 18M**								
SD 18M Animal 1	80 x 80	80 x 80	1	0.33 ± 0.007	1/6	126	6	489
SD 18M Animal 2	80 x 80	80 x 80	1	0.28 ± 0.004	1/6	140	6	559
SD 18M Animal 3	80 x 80	80 x 80	1	0.26 ± 0.002	1/6	168	6	556
SD 18M Animal 4	80 x 80	80 x 80	1	0.36 ± 0.008	1/6	146	6	549

[a]All evaluations were performed using a 60X objective lens (N.A. 1.4; D.F. 0.75µm).
a(frame)' area of the optical dissector counting frame; A(x,y step), x and y step sizes; asf, area sampling fraction [a(frame)/A(x,y step)]; tsf, thickness sampling fraction, calculated by the height of optical dissector divided by section thickness, h/section thickness; ssf, section sampling fraction; $\sum Q^{-}$, counted astrocyte markers
