# Supplementary material for: Spatial memory decline after masticatory deprivation and aging is associated with altered laminar distribution of CA1 astrocytes
Source: BMC Neurosci. 2012 Feb 29;13:23. doi: 10.1186/1471-2202-13-23 (PMC3355053; doi:10.1186/1471-2202-13-23)
Supplement: Additional file 2 — Table S2. Estimated Unilateral Number of Astrocytes (N) With the Coefficient of Error (CE) for the Stratum Radiatum of CA1 of 3-, 6- and 18-Month-Old Female Albino Swiss Mice Fed A Hard Diet (HD) or Soft Diet (SD). [file 1471-2202-13-23-S2.PDF]

Table S2. Experimental Parameters and Optical Fractionator Counting Results in the Stratum Radiatum of CA1 of 3-, 6-, and 18-Month-Old Female Albino Swiss Mice Fed From Weaning With Hard Diet (HD) or Soft Diet (SD).

***Radiatum* - CA1**								
**Subjects [(a)]**	**a(frame) (μm^2)**	**A(x,y step) (μm^2)**	**asf**	**tsf**	**ssf**	**N. of couting frames**	**N. of sections**	**ΣQ^-**
***Hard Diet* / 3M**								
HD 3M Animal 1	80 x 80	80 x 80	1	0.34 ± 0.002	1/6	222	6	560
HD 3M Animal 2	80 x 80	80 x 80	1	0.28 ± 0.004	1/6	153	5	378
HD 3M Animal 3	80 x 80	80 x 80	1	0.27 ± 0.010	1/6	169	6	397
HD 3M Animal 4	80 x 80	80 x 80	1	0.29 ± 0.005	1/6	168	5	452
***Hard Diet* / 6M**								
HD 6M Animal 1	80 x 80	80 x 80	1	0.31 ± 0.012	1/6	166	5	377
HD 6M Animal 2	80 x 80	80 x 80	1	0.39 ± 0.009	1/6	190	6	596
HD 6M Animal 3	80 x 80	80 x 80	1	0.39 ± 0.005	1/6	203	6	705
HD 6M Animal 4	80 x 80	80 x 80	1	0.33 ± 0.004	1/6	188	6	587
***Hard Diet* / 18M**								
HD 18M Animal 1	80 x 80	80 x 80	1	0.29 ± 0.003	1/6	170	6	377
HD 18M Animal 2	80 x 80	80 x 80	1	0.30 ± 0.003	1/6	185	6	432
HD 18M Animal 3	80 x 80	80 x 80	1	0.30 ± 0.003	1/6	142	5	327
HD 18M Animal 4	80 x 80	80 x 80	1	0.30 ± 0.001	1/6	161	5	390
***Soft Diet* / 3M**								
SD 3M Animal 1	80 x 80	80 x 80	1	0.33 ± 0.005	1/6	157	6	379
SD 3M Animal 2	80 x 80	80 x 80	1	0.35 ± 0.011	1/6	152	5	386
SD 3M Animal 3	80 x 80	80 x 80	1	0.32 ± 0.006	1/6	169	5	426

SD 3M Animal 4	80 x 80	80 x 80	1	0.34 ± 0.012	1/6	148	5	500
SD 3M Animal 5	80 x 80	80 x 80	1	0.26 ± 0.006	1/6	136	5	346
***Soft Diet* / 6M**								
SD 6M Animal 1	80 x 80	80 x 80	1	0.35 ± 0.017	1/6	166	5	501
SD 6M Animal 2	80 x 80	80 x 80	1	0.37 ± 0.016	1/6	159	5	468
SD 6M Animal 3	80 x 80	80 x 80	1	0.34 ± 0.015	1/6	144	5	381
SD 6M Animal 4	80 x 80	80 x 80	1	0.28 ± 0.010	1/6	180	6	462
SD 6M Animal 5	80 x 80	80 x 80	1	0.29 ± 0.009	1/6	139	5	329
***Soft Diet* / 18M**								
SD 18M Animal 1	80 x 80	80 x 80	1	0.33 ± 0.006	1/6	185	6	478
SD 18M Animal 2	80 x 80	80 x 80	1	0.28 ± 0.004	1/6	191	6	482
SD 18M Animal 3	80 x 80	80 x 80	1	0.26 ± 0.003	1/6	226	6	515
SD 18M Animal 4	80 x 80	80 x 80	1	0.36 ± 0.008	1/6	182	6	519

[a]All evaluations were performed using a 60X objective lens (N.A. 1.4; D.F. 0.75µm).
a(frame), area of the optical dissector counting frame; A(x,y step), x and y step sizes; asf, area sampling fraction [a(frame)/A(x,y step)]; tsf, thickness sampling fraction, calculated by the height of optical dissector divided by section thickness, h/section thickness; ssf, section sampling fraction; $\sum Q^-$, counted astrocyte markers
