# Supplementary material for: Spatial memory decline after masticatory deprivation and aging is associated with altered laminar distribution of CA1 astrocytes
Source: BMC Neurosci. 2012 Feb 29;13:23. doi: 10.1186/1471-2202-13-23 (PMC3355053; doi:10.1186/1471-2202-13-23)
Supplement: Additional file 3 — Table S3. Estimated Unilateral Number of Astrocytes (N) With the Coefficient of Error (CE) for the Stratum Pyramidale of CA1 of 3-, 6- and 18-Month-Old Female Albino Swiss Mice Fed A Hard Diet (HD) or Soft Diet (SD). [file 1471-2202-13-23-S3.PDF]

Table S3. Experimental Parameters and Optical Fractionator Counting Results in the Stratum Pyramidale of CA1 of 3-, 6-, and 18-Month-Old Female Albino Swiss Mice Fed With Hard Diet (HD) or Soft Diet (SD).

***Stratum Pyramidale* - CA1**								
**Subjects[a]**	**a(frame) (μm^2)**	**A(x,y step) (μm^2)**	**asf**	**tsf**	**ssf**	**N. of couting frames**	**N. of sections**	**ΣQ^-**
***Hard Diet* / 3M**								
HD 3M Animal 1	80 x 80	80 x 80	1	0.34 ± 0.005	1/6	112	6	193
HD 3M Animal 2	80 x 80	80 x 80	1	0.28 ± 0.005	1/6	78	5	121
HD 3M Animal 3	80 x 80	80 x 80	1	0.27 ± 0.009	1/6	79	6	144
HD 3M Animal 4	80 x 80	80 x 80	1	0.29 ± 0.005	1/6	75	5	125
***Hard Diet* / 6M**								
HD 6M Animal 1	80 x 80	80 x 80	1	0.30 ± 0.013	1/6	78	5	123
HD 6M Animal 2	80 x 80	80 x 80	1	0.38 ± 0.009	1/6	102	6	204
HD 6M Animal 3	80 x 80	80 x 80	1	0.38 ± 0.005	1/6	93	6	142
HD 6M Animal 4	80 x 80	80 x 80	1	0.33 ± 0.002	1/6	105	6	208
***Hard Diet* / 18M**								
HD 18M Animal 1	80 x 80	80 x 80	1	0.29 ± 0.004	1/6	85	6	132
HD 18M Animal 2	80 x 80	80 x 80	1	0.29 ± 0.003	1/6	90	6	125
HD 18M Animal 3	80 x 80	80 x 80	1	0.29 ± 0.007	1/6	73	5	120
HD 18M Animal 4	80 x 80	80 x 80	1	0.29 ± 0.000	1/6	79	5	111
***Soft Diet* / 3M**								
SD 3M Animal 1	80 x 80	80 x 80	1	0.33 ± 0.005	1/6	89	6	131
SD 3M Animal 2	80 x 80	80 x 80	1	0.35 ± 0.018	1/6	77	5	120
SD 3M Animal 3	80 x 80	80 x 80	1	0.31 ± 0.005	1/6	73	5	94
SD 3M Animal 4	80 x 80	80 x 80	1	0.32 ± 0.008	1/6	75	5	121
SD 3M Animal 5	80 x 80	80 x 80	1	0.25 ± 0.008	1/6	66	5	80
***Soft Diet* / 6M**								
SD 6M Animal 1	80 x 80	80 x 80	1	0.35 ± 0.019	1/6	81	5	151
SD 6M Animal 2	80 x 80	80 x 80	1	0.37 ± 0.016	1/6	77	5	125
SD 6M Animal 3	80 x 80	80 x 80	1	0.34 ± 0.015	1/6	67	5	92
SD 6M Animal 4	80 x 80	80 x 80	1	0.29 ± 0.008	1/6	77	6	122
SD 6M Animal 5	80 x 80	80 x 80	1	0.28 ± 0.015	1/6	55	5	84
***Soft Diet* / 18M**								
SD 18M Animal 1	80 x 80	80 x 80	1	0.33 ± 0.003	1/6	109	6	174

SD 18M Animal 2	80 x 80	80 x 80	1	0.28 ± 0.003	1/6	91	6	142
SD 18M Animal 3	80 x 80	80 x 80	1	0.26 ± 0.004	1/6	109	6	125
SD 18M Animal 4	80 x 80	80 x 80	1	0.37 ± 0.007	1/6	86	6	174

[a]All evaluations were performed using a 60X objective lens (N.A. 1.4; D.F. 0.75µm). a(frame), area of the optical dissector counting frame; A(x,y step), x and y step sizes; asf, area sampling fraction [a(frame)/A(x,y step)]; tsf, thickness sampling fraction, calculated by the height of optical dissector divided by section thickness, h/section thickness; ssf, section sampling fraction; $\sum Q^-$, counted astrocyte markers.
