# Supplementary material for: Spatial memory decline after masticatory deprivation and aging is associated with altered laminar distribution of CA1 astrocytes
Source: BMC Neurosci. 2012 Feb 29;13:23. doi: 10.1186/1471-2202-13-23 (PMC3355053; doi:10.1186/1471-2202-13-23)
Supplement: Additional file 4 — Table S4. Estimated Unilateral Number of Astrocytes (N) With the Coefficient of Error (CE) for the Stratum Oriens of CA1 of 3-, 6- and 18-Month-Old Female Albino Swiss Mice Fed A Hard Diet (HD) or Soft Diet (SD). [file 1471-2202-13-23-S4.PDF]

Table S4. Experimental Parameters and Optical Fractionator Counting Results in the Stratum Oriens of CA1 of 3-, 6-, and 18-Month-Old Female Albino Swiss Mice Fed With Hard Diet (HD) or Soft Diet (SD).

***Oriens* - CA1**								
**Subjects[a]**	**a(frame) (μm^2)**	**A(x,y step) (μm^2)**	**asf**	**tsf**	**ssf**	**N. of couting frames**	**N. of sections**	**ΣQ$^-$**
***Hard Diet* / 3M**								
HD 3M Animal 1	80 x 80	80 x 80	1	0.34 ± 0.004	1/6	177	6	465
HD 3M Animal 2	80 x 80	80 x 80	1	0.28 ± 0.004	1/6	135	5	348
HD 3M Animal 3	80 x 80	80 x 80	1	0.27 ± 0.008	1/6	130	6	293
HD 3M Animal 4	80 x 80	80 x 80	1	0.29 ± 0.006	1/6	130	5	371
***Hard Diet* / 6M**								
HD 6M Animal 1	80 x 80	80 x 80	1	0.29 ± 0.012	1/6	133	5	309
HD 6M Animal 2	80 x 80	80 x 80	1	0.37 ± 0.011	1/6	141	6	433
HD 6M Animal 3	80 x 80	80 x 80	1	0.37 ± 0.004	1/6	162	6	523
HD 6M Animal 4	80 x 80	80 x 80	1	0.32 ± 0.003	1/6	143	6	401
***Hard Diet* / 18M**								
HD 18M Animal 1	80 x 80	80 x 80	1	0.29 ± 0.004	1/6	144	6	375
HD 18M Animal 2	80 x 80	80 x 80	1	0.29 ± 0.003	1/6	144	6	389
HD 18M Animal 3	80 x 80	80 x 80	1	0.28 ± 0.007	1/6	116	5	290
HD 18M Animal 4	80 x 80	80 x 80	1	0.28 ± 0.002	1/6	131	5	358
***Soft Diet* / 3M**								
SD 3M Animal 1	80 x 80	80 x 80	1	0.33 0.006	1/6	145	6	340
SD 3M Animal 2	80 x 80	80 x 80	1	0.35 ± 0.020	1/6	136	5	341
SD 3M Animal 3	80 x 80	80 x 80	1	0.31 ± 0.003	1/6	126	5	363
SD 3M Animal 4	80 x 80	80 x 80	1	0.33 ± 0.011	1/6	119	5	393
SD 3M Animal 5	80 x 80	80 x 80	1	0.26 ± 0.011	1/6	108	5	252
***Soft Diet* / 6M**								
SD 6M Animal 1	80 x 80	80 x 80	1	0.34 ± 0.019	1/6	132	5	408
SD 6M Animal 2	80 x 80	80 x 80	1	0.36 ± 0.016	1/6	110	5	349
SD 6M Animal 3	80 x 80	80 x 80	1	0.33 ± 0.011	1/6	122	5	327
SD 6M Animal 4	80 x 80	80 x 80	1	0.28 ± 0.007	1/6	135	6	363
SD 6M Animal 5	80 x 80	80 x 80	1	0.28 ± 0.011	1/6	102	5	242
***Soft Diet* / 18M**								
SD 18M Animal 1	80 x 80	80 x 80	1	0.32 ± 0.004	1/6	159	6	453

SD 18M Animal 2	80 x 80	80 x 80	1	0.27 ± 0.003	1/6	163	6	426
SD 18M Animal 3	80 x 80	80 x 80	1	0.26 ± 0.004	1/6	183	6	432
SD 18M Animal 4	80 x 80	80 x 80	1	0.36 ± 0.007	1/6	135	6	399

[a]All evaluations were performed using a 60X objective lens (N.A. 1.4; D.F. 0.75μm). a(frame), area of the optical dissector counting frame; A(x,y step), x and y step sizes; asf, area sampling fraction [a(frame)/A(x,y step)]; tsf, thickness sampling fraction, calculated by the height of optical dissector divided by section thickness, h/section thickness; ssf, section sampling fraction; $\sum Q^-$, counted astrocyte markers.
