# Supplementary material for: Spatial memory decline after masticatory deprivation and aging is associated with altered laminar distribution of CA1 astrocytes
Source: BMC Neurosci. 2012 Feb 29;13:23. doi: 10.1186/1471-2202-13-23 (PMC3355053; doi:10.1186/1471-2202-13-23)
Supplement: Additional file 5 — Table S5. Experimental parameters and optical fractionator counting results in the stratum lacunosum-moleculare of the CA1 of 3-, 6- and 18-month-old female albino Swiss mice fed a hard diet (HD) or soft diet (SD). [file 1471-2202-13-23-S5.PDF]

Table S5. Estimated Unilateral Numbers of Astrocyte (N) With the Coefficient of Error (CE) for the Stratum Lacunosum Moleculare of CA1 of 3-, 6-, and 18-Month-Old Female Albino Swiss Mice Fed A Hard Diet (HD) or Soft Diet (SD).

STRATUM LACUNOSUM MOLECULARE - CA1			
Hard Diet / 3M			
**Subjects**	**N**	**Thickness (μm)**	**CE (Scheaffer)**
HD 3M Animal 1	9650	20.09 ± 0.29	0.06
HD 3M Animal 2	10759	23.22 ± 0.51	0.05
HD 3M Animal 3	9199	25.44 ± 0.92	0.06
HD 3M Animal 4	10490	23.21 ± 0.46	0.06
Mean	10024	22.99 ± 0.54	0.05
SD	725		
CV^2	0.005		
CE^2	0.003		
CE^2/CV^2	0.478		
CVB^2	0.003		
$CVB^2(\%CV^2)$	52.20%		
Soft Diet / 3M			
**Subjects**	**N**	**Thickness (μm)**	**CE (Scheaffer)**
SD 3M Animal 1	8318	20.74 ± 0.40	0.05
SD 3M Animal 2	8179	19.90 ± 0.36	0.06
SD 3M Animal 3	7535	21.43 ± 0.26	0.06
SD 3M Animal 4	10429	20.67 ± 0.69	0.05
SD 3M Animal 5	9641	26.64 ± 0.68	0.06
Mean	8820	21.88 ± 0.48	0.06
SD	1180		
CV^2	0.018		
CE^2	0.003		
CE^2/CV^2	0.140		
CVB^2	0.015		
$CVB^2(\%CV^2)$	86.03%		
Hard Diet / 6M			
**Subjects**	**N**	**Thickness (μm)**	**CE (Scheaffer)**
HD 6M Animal 1	9900	21.74 ± 0.68	0.05
HD 6M Animal 2	11888	17.50 ± 0.36	0.05
HD 6M Animal 3	12019	18.40 ± 0.16	0.05
HD 6M Animal 4	12895	21.26 ± 0.33	0.04
Mean	11675	19.72 ± 0.38	0.05
SD	1265		

CV^2	0.012		
CE^2	0.002		
CE^2/CV^2	0.136		
CVB^2	0.010		
$CVB^2(\%CV^2)$	86.37%		
Soft Diet / 6M			
**Subjects**	**N**	**Thickness (μm)**	**CE (Scheaffer)**
SD 6M Animal 1	11613	20.12 ± 0.89	0.05
SD 6M Animal 2	9797	19.03 ± 0.76	0.05
SD 6M Animal 3	9433	21.15 ± 1.05	0.05
SD 6M Animal 4	9575	24.31 ± 0.68	0.06
SD 6M Animal 5	10534	24.85 ± 0.76	0.05
Mean	10190	21.89 ± 0.90	0.05
SD	901		
CV^2	0.008		
CE^2	0.003		
CE^2/CV^2	0.320		
CVB^2	0.005		
$CVB^2(\%CV^2)$	68.02%		
Hard Diet / 18M			
**Subjects**	**N**	**Thickness (μm)**	**CE (Scheaffer)**
HD 18M Animal 1	8870	23.65 ± 0.27	0.05
HD 18M Animal 2	9153	23.37 ± 0.25	0.06
HD 18M Animal 3	6806	23.59 ± 0.45	0.06
HD 18M Animal 4	9386	23.7 ± 0.38	0.06
Mean	8553	23.57 ± 0.33	0.06
SD	1183		
CV^2	0.019		
CE^2	0.003		
CE^2/CV^2	0.131		
CVB^2	0.017		
$CVB^2(\%CV^2)$	86.93%		
Soft Diet / 18M			
**Subjects**	**N**	**Thickness (μm)**	**CE (Scheaffer)**
SD 18M Animal 1	9052	21.21 ± 0.46	0.06
SD 18M Animal 2	11710	24.32 ± 0.38	0.05
SD 18M Animal 3	12714	26.62 ± 0.29	0.05
SD 18M Animal 4	9171	19.43 ± 0.43	0.05
Mean	10661	22.89 ± 0.39	0.05

SD	1837		
CV^2	0.030		
CE^2	0.003		
CE^2/CV^2	0.084		
CVB^2	0.027		
$CVB^2(\%CV^2)$	91.57%		

$CVB^2 = CV^2 – CE^2$ (CV, coefficient of variation; CVB, biological coefficient of variation; CE, coefficient of error). N = number of astrocytes; Mean = mean numbers in each group; SD, standard deviation; 3M, 6M, and 18M indicate 3 months old, 6 months old, and 18 months old, respectively.
