# Supplementary material for: Spatial memory decline after masticatory deprivation and aging is associated with altered laminar distribution of CA1 astrocytes
Source: BMC Neurosci. 2012 Feb 29;13:23. doi: 10.1186/1471-2202-13-23 (PMC3355053; doi:10.1186/1471-2202-13-23)
Supplement: Additional file 6 — Table S6. Experimental Parameters and Optical Fractionator Counting Results in the Stratum Radiatum of CA1 of 3-, 6- and 18-Month-Old Female Albino Swiss Mice Fed A Hard Diet (HD) or Soft Diet (SD). [file 1471-2202-13-23-S6.PDF]

Table S6. Estimated Unilateral Numbers of Astrocyte (N) With the Coefficient of Error (CE) for the Stratum Radiatum of CA1 of 3-, 6-, and 18-Month-Old Female Albino Swiss Mice Fed A Hard Diet (HD) or Soft Diet (SD).

STRATUM RADIATUM - CA1			
Hard Diet / 3M			
**Subjects**	**N**	**Thickness (µm)**	**CE (Scheaffer)**
HD 3M Animal 1	9624	20.16 ± 0.13	0.04
HD 3M Animal 2	7810	24.25 ± 0.37	0.05
HD 3M Animal 3	8812	25.24 ± 1.06	0.05
HD 3M Animal 4	9079	23.47 ± 0.44	0.04
Mean	8831	23.28 ± 0.50	0.04
SD	760		
CV^2	0.007		
CE^2	0.002		
CE^2/CV^2	0.216		
CVB^2	0.006		
$CVB^2(\%CV^2)$	78.39%		
Soft Diet / 3M			
**Subjects**	**N**	**Thickness (µm)**	**CE (Scheaffer)**
SD 3M Animal 1	6803	20.92 ± 0.30	0.05
SD 3M Animal 2	6446	19.68 ± 0.55	0.05
SD 3M Animal 3	7822	21.66 ± 0.40	0.04
SD 3M Animal 4	8907	20.65 ± 0.76	0.04
SD 3M Animal 5	8086	27.05 ± 0.77	0.05
Mean	7612	21.99 ± 0.55	0.05
SD	994		
CV^2	0.017		
CE^2	0.002		
CE^2/CV^2	0.094		
CVB^2	0.015		
$CVB^2(\%CV^2)$	90.61%		
Hard Diet / 6M			
**Subjects**	**N**	**Thickness (µm)**	**CE (Scheaffer)**
HD 6M Animal 1	7203	22.05 ± 0.82	0.05
HD 6M Animal 2	9060	17.89 ± 0.45	0.04
HD 6M Animal 3	10763	17.92 ± 0.25	0.03

HD 6M Animal 4	10345	20.65 ± 0.27	0.03
Mean	9342	19.62 ± 0.44	0.04
SD	1600		
CV^2	0.029		
CE^2	0.001		
CE^2/CV^2	0.031		
CVB^2	0.028		
$CVB^2(\%CV^2)$	96.93%		
Soft Diet / 6M			
**Subjects**	**N**	**Thickness (µm)**	**CE (Scheaffer)**
SD 6M Animal 1	8495	19.98 ± 1.01	0.04
SD 6M Animal 2	7384	18.67 ± 0.91	0.04
SD 6M Animal 3	6671	20.68 ± 1.07	0.05
SD 6M Animal 4	6799	24.05 ± 0.72	0.05
SD 6M Animal 5	9772	24.36 ± 0.93	0.04
Mean	7824	21.54 ± 0.92	0.04
SD	1305		
CV^2	0.028		
CE^2	0.002		
CE^2/CV^2	0.058		
CVB^2	0.026		
$CVB^2(\%CV^2)$	94.24%		
Hard Diet / 18M			
**Subjects**	**N**	**Thickness (µm)**	**CE (Scheaffer)**
HD 18M Animal 1	7483	23.28 ± 0.37	0.05
HD 18M Animal 2	8483	23.12 ± 0.24	0.05
HD 18M Animal 3	6534	23.33 ± 0.40	0.05
HD 18M Animal 4	7731	23.19 ± 0.08	0.05
Mean	7557	23.23 ± 0.27	0.05
SD	803		
CV^2	0.011		
CE^2	0.002		
CE^2/CV^2	0.142		
CVB^2	0.010		
$CVB^2(\%CV^2)$	85.82%		
Soft Diet / 18M			

Subjects	N	Thickness (μm)	CE (Scheaffer)
SD 18M Animal 1	8606	21.21 ± 0.42	0.04
SD 18M Animal 2	10059	24.32 ± 0.34	0.04
SD 18M Animal 3	11735	26.72 ± 0.36	0.04
SD 18M Animal 4	8567	19.13 ± 0.44	0.04
Mean	9741	22.84 ± 0.39	0.04
SD	1499		
CV^2	0.024		
CE^2	0.002		
CE^2/CV^2	0.068		
CVB^2	0.022		
$CVB^2(\%CV^2)$	93.24%		

$CVB^2 = CV^2 - CE^2$ (CV, coefficient of variation; CVB, biological coefficient of variation; CE, coefficient of error). N = number of astrocytes; Mean = mean numbers in each group; SD, standard deviation; 3M, 6M, and 18M indicate 3 months old, 6 months old, and 18 months old, respectively.
