# Supplementary material for: Spatial memory decline after masticatory deprivation and aging is associated with altered laminar distribution of CA1 astrocytes
Source: BMC Neurosci. 2012 Feb 29;13:23. doi: 10.1186/1471-2202-13-23 (PMC3355053; doi:10.1186/1471-2202-13-23)
Supplement: Additional file 7 — Table S7. Experimental Parameters and Optical Fractionator Counting Results in the Stratum Pyramidale of CA1 of 3-, 6- and 18-Month-Old Female Albino Swiss Mice Fed A Hard Diet (HD) or Soft Diet (SD). [file 1471-2202-13-23-S7.PDF]

Table S7. Estimated Unilateral Numbers of Astrocytes (N) With the Coefficient of Error (CE) for the Stratum Pyramidale of CA1 of 3-, 6-, and 18-Month-Old Female Albino Swiss Mice Fed A Hard Diet (HD) or Soft Diet (SD).

STRATUM PYRAMIDALE - CA1			
Hard Diet / 3M			
**Subjects**	**N**	**Thickness (µm)**	**CE (Scheaffer)**
HD 3M Animal 1	3400	20.45 ± 0.32	0.05
HD 3M Animal 2	2592	25.01 ± 0.50	0.07
HD 3M Animal 3	2547	25.77 ± 0.82	0.07
HD 3M Animal 4	2565	23.95 ± 0.47	0.07
Mean	2775	23.79 ± 0.52	0.07
SD	416		
CV2	0.022		
CE2	0.004		
CE2/CV2	0.160		
CVB2	0.019		
CVB2(%CV2)	83.98%		
Soft Diet / 3M			
**Subjects**	**N**	**Thickness (µm)**	**CE (Scheaffer)**
SD 3M Animal 1	2333	20.75 ± 0.34	0.07
SD 3M Animal 2	1979	20.04 ± 0.84	0.08
SD 3M Animal 3	1794	22.47 ± 0.37	0.08
SD 3M Animal 4	2243	21.51 ± 0.58	0.07
SD 3M Animal 5	1892	27.12 ± 0.96	0.07
Mean	2048	22.38 ± 0.62	0.07
SD	230		
CV^2	0.013		
CE^2	0.005		
CE^2/CV^2	0.389		
CVB^2	0.008		
$CVB^2(\%CV^2)$	61.14%		
Hard Diet / 6M			
**Subjects**	**N**	**Thickness (µm)**	**CE (Scheaffer)**
HD 6M Animal 1	2454	22.84 ± 0.94	0.09
HD 6M Animal 2	3175	18.26 ± 0.46	0.06

HD 6M Animal 3	2194	18.07 ± 0.24	0.07
HD 6M Animal 4	3722	20.86 ± 0.18	0.05
Mean	1886	20.00 ± 0.45	0.07
SD	694		
CV^2	0.135		
CE^2	0.004		
CE^2/CV^2	0.027		
CVB^2	0.132		
$CVB^2(\%CV^2)$	97.34%		
Soft Diet / 6M			
**Subjects**	**N**	**Thickness (μm)**	**CE (Scheaffer)**
SD 6M Animal 1	2582	20.07 ± 1.12	0.07
SD 6M Animal 2	1954	18.75 ± 0.94	0.09
SD 6M Animal 3	1628	20.73 ± 1.00	0.07
SD 6M Animal 4	1767	14.65 ± 1.18	0.09
SD 6M Animal 5	2365	24.27 ± 0.72	0.06
Mean	2059	19.69 ± 0.99	0.07
SD	403		
CV^2	0.038		
CE^2	0.005		
CE^2/CV^2	0.128		
CVB^2	0.033		
$CVB^2(\%CV^2)$	87.20%		
Hard Diet / 18M			
**Subjects**	**N**	**Thickness (μm)**	**CE (Scheaffer)**
HD 18M Animal 1	2657	23.51 ± 0.34	0.08
HD 18M Animal 2	2503	23.61 ± 0.30	0.07
HD 18M Animal 3	2481	23.83 ± 0.63	0.08
HD 18M Animal 4	2304	24.04 ± 0.06	0.08
Mean	2486	23.74 ± 0.33	0.08
SD	144		
CV^2	0.003		
CE^2	0.005		
CE^2/CV^2	1.460		
CVB^2	-0.002		

$CVB^2(\%CV^2)$	-46.04%		
Soft Diet / 18M			
**Subjects**	**N**	**Thickness (μm)**	**CE (Scheaffer)**
SD 18M Animal 1	3171	21.25 ± 0.22	0.07
SD 18M Animal 2	3035	24.98 ± 0.30	0.07
SD 18M Animal 3	2844	26.8 ± 0.46	0.07
SD 18M Animal 4	2773	18.68 ± 0.35	0.06
Mean	2955	22.92 ± 0.33	0.07
SD	181		
CV^2	0.004		
CE^2	0.004		
CE^2/CV^2	0.960		
CVB^2	0.000		
$CVB^2(\%CV^2)$	4.04%		

$CVB^2 = CV^2 – CE^2$ (CV, coefficient of variation; CVB, biological coefficient of variation; CE, coefficient of error). N = number of astrocytes; Mean = mean numbers in each group; SD, standard deviation; 3M, 6M, and 18M indicate 3 months old, 6 months old, and 18 months old, respectively.
