# Supplementary material for: Spatial memory decline after masticatory deprivation and aging is associated with altered laminar distribution of CA1 astrocytes
Source: BMC Neurosci. 2012 Feb 29;13:23. doi: 10.1186/1471-2202-13-23 (PMC3355053; doi:10.1186/1471-2202-13-23)
Supplement: Additional file 8 — Table S8. Experimental Parameters and Optical Fractionator Counting Results in the Stratum Oriens of CA1 of 3-, 6- and 18-Month-Old Female Albino Swiss Mice Fed A Hard Diet (HD) or Soft Diet (SD). [file 1471-2202-13-23-S8.PDF]

Table S8. Estimated Unilateral Numbers of Astrocytes (N) With the Coefficient of Error (CE) for the Stratum Oriens of CA1 of 3-, 6-, and 18-Month-Old Female Albino Swiss Mice Fed A Hard Diet (HD) or Soft Diet (SD).

ORIENS - CA1			
Hard Diet / 3M			
**Subjects**	**N**	**Thickness (µm)**	**CE (Scheaffer)**
HD 3M Animal 1	8152	20.46 ± 0.28	0.04
HD 3M Animal 2	7420	25.04 ± 0.40	0.05
HD 3M Animal 3	6448	25.41 ± 0.74	0.06
HD 3M Animal 4	7454	23.43 ± 0.48	0.05
Mean	7368	23.59 ± 0.52	0.05
SD	700		
CV2	0.009		
CE2	0.003		
CE2/CV2	0.277		
CVB2	0.007		
CVB2(%CV2)	72.30%		
Soft Diet / 3M			
**Subjects**	**N**	**Thickness (µm)**	**CE (Scheaffer)**
SD 3M Animal 1	6226	21.26 ± 0.39	0.05
SD 3M Animal 2	5661	19.96 ± 0.97	0.06
SD 3M Animal 3	6800	22.05 ± 0.25	0.05
SD 3M Animal 4	7193	21.33 ± 0.78	0.05
SD 3M Animal 5	5839	26.95 ± 1.23	0.06
Mean	6344	22.31 ± 0.72	0.06
SD	644		
CV^2	0.010		
CE^2	0.003		
CE^2/CV^2	0.243		
CVB^2	0.008		
$CVB^2(\%CV^2)$	75.73%		
Hard Diet / 6M			
**Subjects**	**N**	**Thickness (µm)**	**CE (Scheaffer)**
HD 6M Animal 1	6327	23.83 ± 0.92	0.05
HD 6M Animal 2	6887	18.81 ± 0.58	0.05

HD 6M Animal 3	8395	18.85 ± 0.24	0.04
HD 6M Animal 4	7347	21.45 ± 0.21	0.05
Mean	7238.807143	20.73 ± 0.48	0.05
SD	876		
CV^2	0.015		
CE^2	0.002		
CE^2/CV^2	0.109		
CVB^2	0.013		
$CVB^2(\%CV^2)$	89.07%		
Soft Diet / 6M			
**Subjects**	**N**	**Thickness (µm)**	**CE (Scheaffer)**
SD 6M Animal 1	7238	20.71 ± 1.18	0.05
SD 6M Animal 2	5681	19.34 ± 1.01	0.05
SD 6M Animal 3	5878	21.28 ± 0.82	0.05
SD 6M Animal 4	5269	25.11 ± 1.00	0.05
SD 6M Animal 5	7946	25.05 ± 0.70	0.05
Mean	6402	22.29 ± 0.94	0.05
SD	1135		
CV^2	0.031		
CE^2	0.003		
CE^2/CV^2	0.080		
CVB^2	0.029		
$CVB^2(\%CV^2)$	92.04%		
Hard Diet / 18M			
**Subjects**	**N**	**Thickness (µm)**	**CE (Scheaffer)**
HD 18M Animal 1	7789	24.14 ± 0.32	0.05
HD 18M Animal 2	7969	24.02 ± 0.27	0.05
HD 18M Animal 3	6170	24.77 ± 0.71	0.07
HD 18M Animal 4	7435	24.27 ± 0.20	0.06
Mean	7340	24.30 ± 0.37	0.06
SD	811		
CV^2	0.012		
CE^2	0.003		
CE^2/CV^2	0.205		
CVB^2	0.010		

$CVB^2(\%CV^2)$	79.52%		
Soft Diet / 18M			
**Subjects**	**N**	**Thickness (μm)**	**CE (Scheaffer)**
SD 18M Animal 1	8478	21.86 ± 0.30	0.05
SD 18M Animal 2	9325	25.50 ± 0.38	0.05
SD 18M Animal 3	9841	26.92 ± 0.45	0.05
SD 18M Animal 4	6521	19.09 ± 0.37	0.04
Mean	8541	23.34 ± 0.37	0.05
SD	1459		
CV^2	0.029		
CE^2	0.002		
CE^2/CV^2	0.055		
CVB^2	0.028		
$CVB^2(\%CV^2)$	94.51%		

$CVB^2 = CV^2 – CE^2$ (CV, coefficient of variation; CVB, biological coefficient of variation; CE, coefficient of error). N = number of astrocytes; Mean = mean numbers in each group; SD, standard deviation; 3M, 6M, and 18M indicate 3 months old, 6 months old, and 18 months old, respectively.
